# Adapting the Accelerated Solvent Extraction Method for Resin and Rubber Determination in Guayule Using the BÜCHI Speed Extractor

**DOI:** 10.3390/molecules26010183

**Published:** 2021-01-01

**Authors:** Juana Rozalén, María de las Mercedes García-Martínez, María Engracia Carrión, Manuel Carmona, Horacio López-Córcoles, Katrina Cornish, Amaya Zalacain

**Affiliations:** 1 Catedra de Química Agrícola, Escuela Técnica Superior de Ingenieros Agrónomos y de Montes, Universidad de Castilla-La Mancha, Campus Universitario s/n, 02071 Albacete, Spain; juana.rozalen@uclm.es (J.R.); Amaya.Zalacain@uclm.es (A.Z.); 2 Food Quality Research Group, Institute for Regional Development (IDR), Universidad de Castilla-La Mancha, 02071 Albacete, Spain; mariamercedes.garcia@uclm.es; 3 Food Technology Lab, School of Architecture, Engineering and Design, Universidad Europea de Madrid, C/Tajo s/n, 28670 Villaviciosa de Odón, Spain; mengracia@linkidi.com; 4 Research Department, Instituto Técnico Agronómico Provincial de Albacete (ITAP), Parque Empresarial Campollano, 2^a^ Avenida, 61, 02007 Albacete, Spain; hlc.itap@dipualba.es; 5 Departments of Horticulture and Crop Science and of Food Agricultural and Biological Engineering, College of Food, Agricultural and Environmental Sciences, The Ohio State University, Wooster, OH 44691, USA; cornish.19@osu.edu

**Keywords:** guayule, accelerated solvent extraction (ASE), natural rubber, resin

## Abstract

Guayule (*Parthenium argentatum* Gray) is a promising alternative source to *Hevea brasiliensis* for the production of natural rubber, which can reach levels of 8–9% under industrialized farming conditions. The most common method for determining rubber concentration is by accelerated solvent extraction (ASE), a technique developed by the Dionex Corporation and almost exclusively performed with the Dionex ASE-200 or 350 systems. Herein, it is sought to apply and adapt the most common methods used in the literature for the Dionex system to another extraction platform, the BÜCHI Speed Extractor E-914. Results showed that using a sand sandwich method to confine the sample in the center and exploiting a larger cell volume (80 mL) for extraction prevents the occurrence of overpressure and problems with clogging. Under optimized conditions, the coefficient of variation was <15% for both resin quantification for samples containing 5.0–15.8% of resin and for rubber quantification for samples with 1.7–10.3% rubber content. The extraction time for resin (2 cycles of 5 min each) was smaller than for rubber (2 cycles of 20 min each). It would be interesting to carry out interlaboratory comparisons to standardize the method at an international level.

## 1. Introduction

Guayule (*Parthenium argentatum* Gray) is an arid-adapted, low-input perennial shrub native to Mexico and Southern Texas that has received considerable attention as an alternative source of natural rubber. It has the potential to replace natural rubber, which is tapped as latex from Hevea trees (*Hevea brasiliensis*) grown in tropical regions, primarily Southeast Asia. In addition to natural rubber, guayule also has a resin fraction containing many identified terpenoid compounds [1], as well as fatty acid triglycerides, lipids, pigments, and other acetone-soluble materials [2] that are amenable for exploitation. The isolation/extraction of both the rubber and resin fractions in guayule tissue is performed by solid–liquid extraction using organic solvents [3,4]. The prevalent accelerated solvent extraction (ASE) quantification method employs sequential extraction, first with acetone to extract the resin fraction and then with hexane to extract the high molecular weight rubber [3,4], and yields are then gravimetrically determined. Other solvent-based extraction techniques such as Soxhlet extraction (used for more than a century) or homogenization [5,6,7] are not used as often as they were because they are now known to degrade natural rubber and to overestimate the yield of resin [3,8]. The development of ASE, a pressurized liquid extraction, circumvents these issues. Although partial rubber degradation does occur, this is limited by the rapid extraction time in a pressurized nitrogen atmosphere, and since the rubber is extracted after the resin fraction this degradation does not affect rubber quantification. An important additional advantage of ASE over traditional extraction methods is the reduced handling of organic solvents (through automation) and the increased extraction speed and efficiency through the use of high pressure in combination with high temperature [9,10]. The most widely used measurement platforms in guayule research are the Dionex ASE 200 or 350 extractors (Dionex Corp., Bannockburn, IL, USA) [3,8,9,11,12], but other new systems and analytical methods start to be applied to the guayule [13,14]. It is necessary to test and standardize them to allow direct comparisons among methods and to determine if they are more versatile for the still laborious task of analyzing the resin and rubber content of the guayule.

In the present study, the development of protocols for gravimetric determination of resin and rubber content in guayule stems using the BÜCHI Speed Extractor E-914 platform (Postfach, Switzerland) is described. In the process of developing the protocol, equipment configuration, sample quantity and ratio to solvent, as well as extraction time in each cycle have been addressed in an attempt to address the challenges typically encountered when translating studies from the literature, including equipment clogging and reproducibility issues.

## 2. Results and Discussion

The extraction conditions for guayule in studies with the Dionex ASE instrument are variable, for example, 1, 1.45, or 1.5 g of guayule in an 11 mL cell, [3,8,9,10], and 5 g in a 22 mL cell [11]. Additionally, based on previous knowledge and experience with conventional homogenizing or Soxhlet extraction, the use of acetone at a temperature of 40 °C and a pressure of 103 bar is commonly used to perform a three-cycle protocol (20 min per cycle) with the Dionex ASE instruments to extract resin [3,8,9,11]. For rubber extraction, different strong apolar solvents including cyclohexane, pentane, and hexane have been tested, and the most common option is hexane at 100–140 °C and 103 bar [3,8,9,11].

### 2.1. Sample Preparation and Equipment Adjustment

**Figure 1:**
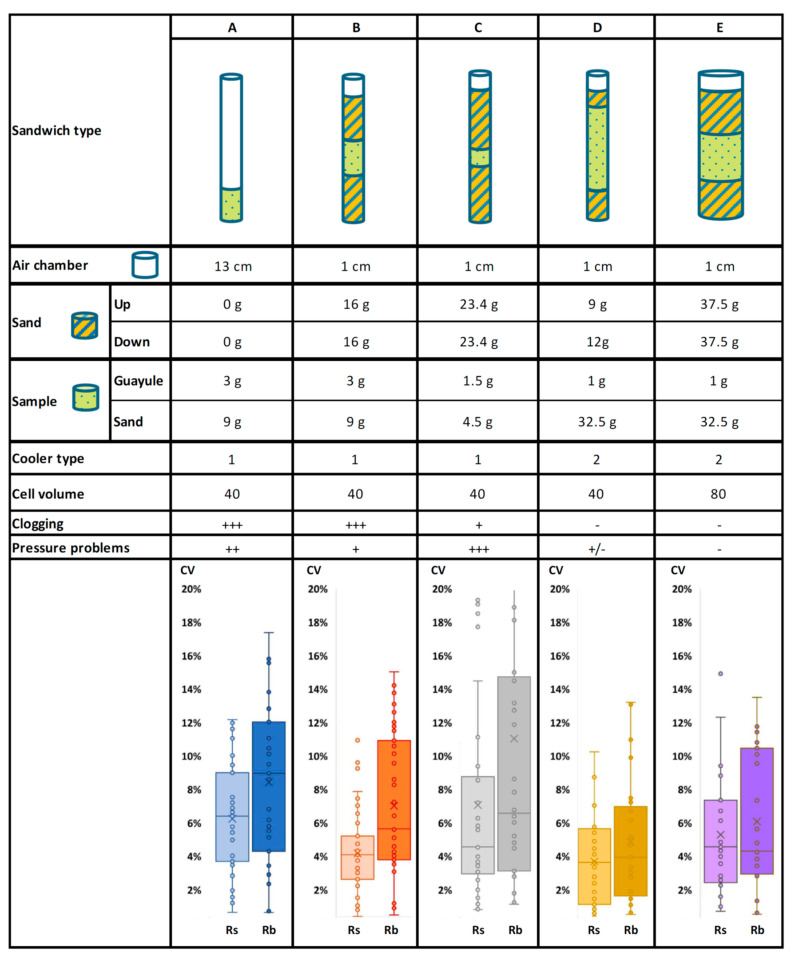
Overall design of the testing conditions and the coefficients of variation (CV) for resin (Rs) and rubber (Rb) extractions from 30 guayule samples extracted by BÜCHI accelerated solvent extraction (BASE) using five different sample preparation processes (**A**–**E**). The fixed parameters for resin were acetone as solvent, 3 cycles of 20 min at 40 °C and 100 bar and for rubber, hexane as solvent, 3 cycles of 20 min at 120 °C and 100 bar. Pressure and clogging problems are represented using a scale from +++ (the highest value, many problems) to—(the lower value or absence of problems).

The BÜCHI Speed Extractor E-914 is equipped with extraction cells of different volumes: 40, 80, and 120 mL. First, the smallest cell was used to test the conditions reported in the literature, which are represented as condition A in Figure 1. Each guayule sample was mixed with sand as a dispersing agent to avoid blocking the filter disk and/or the metal frit by fine guayule particles. The initial parameters were modified according to the results as the process was optimized.

Condition A caused complete blockage of the BÜCHI system due to high-pressure problems and clogging, and the equipment had to be dismantled and cleaned. If the sample is too large or too concentrated, there is a greater probability that the unit will be clogged with resin and/or rubber. Additionally, because of the large amount of free space in the extraction cell and the high-pressure conditions, the sample may move beyond the cell and contribute to the clogging. Thus, following the previously reported ASE conditions (sample/cell volume), the Speed Extractor E-914 was easily clogged, and sample reproducibility was inconsistent. The protocol was then adapted to address these issues (condition B in Figure 1).

Condition B (Figure 1) was established to improve sample packing by sandwiching the sample between the sand beds and reducing the void volume at the top. These conditions reduced pressure problems, but clogging issues remained. However, condition B had clearly better reproducibility of resin extraction data, not so evident for rubber extraction (see the CVs in the Figure 1 boxplots).

Conditions C and D were designed to address clogging issues by reducing the amount of sample and maintaining the sand beds and led to a significant improvement in equipment performance. While the CVs for resin and rubber determination did not improve under condition C, condition D resulted in lower CVs (Figure 1).

Equipment blockage due to high pressure continued to occur intermittently during routine analysis. It was tested the use of a larger extraction cell to prevent this occurrence (condition E) as more diluted extracts ensue. Condition E used the same amount of guayule sample as condition D, but in a larger extraction cell volume (80 mL) with a proportionally scaled sand bed structure. A drawback to this set-up is the higher solvent consumption, although it was recovered by evaporation and condensation before the gravimetric quantification of resins and rubber. The Speed Extractor E-914 used under these conditions did not suffer from any pressure or clogging blockage during extended periods of use. Both resin and rubber quantification (condition E) had low CVs between samples, although in the case of rubber determination, a higher CV was observed when compared with condition D (40 mL cell). The best CV data corresponded to sandwich D and E, but condition E was finally selected as optimal for this equipment because there were no pressure problems, as occurred in condition D. The larger 120 mL cell was not considered as it would require even greater solvent volumes.

### 2.2. Cycle Adjustment

**Figure 2:**
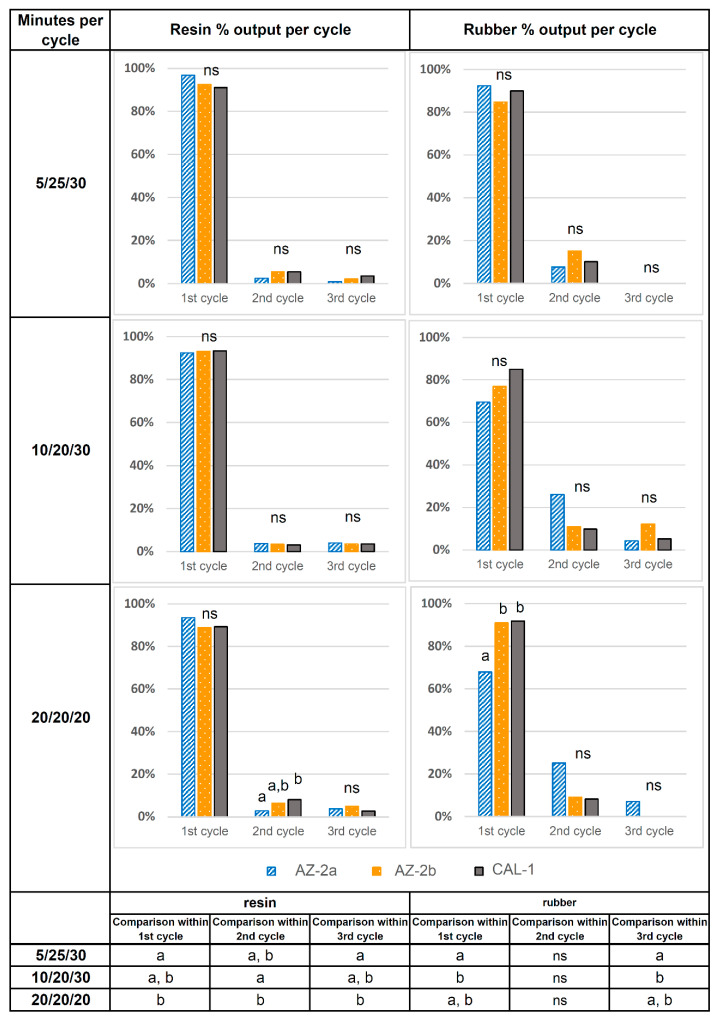
Testing the 3 different cycle schemes in a total extraction time of 60 min for resin extraction and then a total extraction time of 60 min for rubber. Gravimetric yield for resin and rubber in each single stage from the total extracted. Comparisons were made between 2 accessions (AZ-2 and CAL1), with the AZ-2 accession originating from two different plots (AZ-2a and AZ-2b). Different letters in the graph denote significant differences between accessions (AZ-2a, AZ-2b, and CAL1) within cycles, while different letters in columns of the table below denote significant differences between extraction time within the same extraction cycle, both at a 95% level of confidence. ns = non-significant.

Once sample preparation was considered optimal (condition E, above), it was next considered the extraction time. Cycle length conditions used are typically 20 min in three consecutive steps of 20/20/20 [11], although some alternative extraction protocols using a lower number of cycles (1–2) and shorter cycle duration (5–10 min) have produced good results [2]. Thus, three different cycle times were investigated (20/20/20; 10/20/30; 5/25/30) while maintaining the total extraction time at 60 min. The experiment was repeated twice for every sample to reduce variability among the guayule varieties. The monitoring of the extraction process was performed in a differentiated way for each of the stages, separating and quantifying the extracted resins and rubber (Figure 2) at each stage.

With regard to resin extraction, significant differences were found in the percentage extracted between the first cycle of 5 or 20 min in the different extraction schemes, with slightly higher extraction percentages at 5 min. Greater than 90% of the resin present in the samples was extracted in the first cycle, and the two varieties tested (AZ-2 and CAL-1) behaved similarly (Figure 2) with a similar average resin content (8.8–9.1%). Most of the resin was extracted (92–96% of the total yield) in the first and second cycle, which is similar to a previous study where 88–95% yield was achieved by a single extraction [3]. The purpose of starting with a short cycle time of 5 min was to reduce the amount of resin obtained at this first stage to support the previous efforts to prevent clogging. Since a single extraction is not considered sufficient [2], it was preferable to distribute the yield between the first and second extraction. However, 5 min was as efficient as the longer extraction times.

The behavior between the varieties changed slightly during the extraction of natural rubber. One of the plots of AZ-2 (AZ-2a, Figure 2) showed a significant decrease in extraction yield when the duration of the first extraction stage was extended to 20 min. Significant differences were observed between the 5 and 10 min extraction times, although the latter was not different from the results of the 20 min extraction. A shorter extraction time provided a higher proportion of rubber in the first extraction. It is difficult to explain this behavior, but it could be related to the fact that there is a slight increase in rubber content when extraction with the apolar solvent is preceded by multiple short extractions with acetone at 40 °C [8].

**Figure 3:**
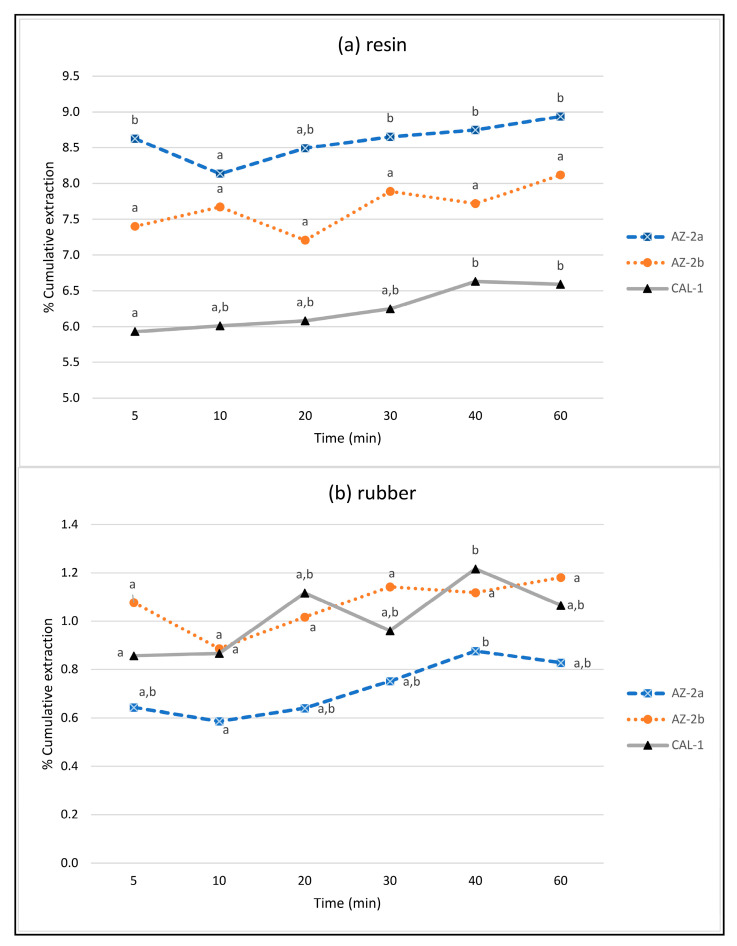
Cumulative resin (**a**) and rubber (**b**) extraction yield depending on the extraction time without considering the number of cycles. Comparisons were made between 2 accessions (AZ-2 and CAL1), with the AZ-2 accession originating from two different plots (AZ-2a and AZ-2b). Different letters within the same accession and product extracted denote statistically significant differences between the yields at any extraction time at a 95% level of confidence.

To determine the best conditions in each case—resin and rubber—they were analyzed the times independently of the number of cycles (Figure 3). The results obtained were not the same for all the accessions, justifying our efforts to examine more than one accession and even different plantations of the same accession. It is noteworthy that of the three samples tested the AZ-2 sample had the highest resin content in the field (8.1–8.9% depending on the extraction conditions) but, on the contrary, it was the one with the lowest natural rubber content (0.6–0.9%) of the three samples tested.

For the resin extraction, the AZ-2 accession in plot b behaved similarly with regard to the amount of resin extracted under all of the conditions tested (Figure 3a). In the other plot of the same accession (AZ-2a), there was no difference between extracting the guayule for 5 or 60 min, although the extraction in a single step of 10 min produced a lower yield and showed significant differences compared with 5 min and extraction times longer than 30 min.

Finally, for the CAL-1 accession, there was no difference between the 10 min extraction and any of the longer times, including the overall 60 min extraction, but differences for resin extracted were observed when compared to larger extraction times (40 or 60 min) with the 5 min extraction.

The cumulative extraction results (Figure 3a) indicate that accurate quantification of resin can be achieved by a two sequential 5 min extraction under sample condition E. Longer extraction times did not significantly affect the results for the two accessions tested. Our results agree with those obtained by Pearson et al. [8] using the Dionex ASE system, where only two static stages of 5 min are sufficient to achieve complete extraction of the resins.

Extraction of natural rubber required longer extraction times than for resin. Again, the yield obtained from the AZ-2b accession did not vary between the extraction conditions (Figure 3a,b). In the other two cases (AZ-2a and CAL-1), however, there were significant differences between the 10 and 40 min (20/20 min) extractions, but no differences between the 20, 30 and 60 min extractions (Figure 3b). Pearson et al. [8] found that extending the time from 5 to 20 min increased the amount of rubber extracted by 30%. In our case, it is concluded that the best procedure is to use two 20 min cycles for a total extraction time of 40 min, with which even greater results are obtained than with three cycles of the same duration, even if there are no statistically significant differences.

In summary, with the sample configuration optimized in this work, it is proposed to reduce the number of cycles from three to two and the extraction time of the acetone stage from 20 to 5 min when using the BÜCHI E-914 for the extraction and gravimetric quantification of the resin and rubber content, as compared with the most recent literature using the Dionex equipment [12]. Although this time reduction can increase the workflow in the laboratory, there are some limitations, and each ASE system on the market has its advantages and disadvantages. The Dionex ASE 350 has greater independence; once programmed, it can work operator-free for long periods by its 24-sample capacity carousel. However, it is only capable of extracting one sample at a time, which means that due to the high thermal inertia of the extraction cells, all the samples must be extracted from the carousel with acetone (40 °C) before raising the temperature (100–140 °C) and starting the extraction of natural rubber with hexane. If an attempt is made to extract the same sample consecutively, it is common for the equipment to shut down for safety reasons because of its inability to cool down quickly [8]. The possible impact in rubber yield of having the guayule sample “wet” with acetone (despite the purge step that accompanies the discharge of the solvent) for several hours before extraction with the hexane is unknown. The BÜCHI equipment can extract four samples simultaneously (there is another system with six extraction cells, the E-916), and so the period that elapses between the acetone the hexane extraction is very short. It does, however, require an operator to remove the flasks where the resins have been collected during the extraction with acetone and replace them with empty ones to collect the natural rubber dissolved in the hexane. In addition, the optimized conditions for guayule established in this study require much higher volumes of solvents than the Dionex methods. This would not be a problem a priori if both solvents are recovered and reused, as is done in our laboratory. Indeed, this raises an opportunity to incorporate similar solvent recapture in the Dionex system. Even though a smaller volume of solvents is used, the common approach is not to recover them but to evaporate them in a fume hood in Petri dishes/plates, and give the tared plates a final drying in an oven before they are reweighed.

Comparing both systems, it was estimated that in a working week, the maximum sample throughput would be 26 for the Dionex ASE 200 and 25 for the BÜCHI E-914 (experiment including duplicates of each sample in both cases). Considering that the Dionex extraction can be performed over 24 h (operator-free) and the BÜCHI cannot, it has 4 extraction channels and is estimated to work 12 h in consecutive shifts of the lab personnel. It would be interesting to perform a comparative study of both methods, not only to determine the precision and establish a standard of quantification of resins and natural rubber in guayule but also to estimate the economic cost per sample by considering the necessary labor cost, investment, consumables, and electrical consumption. As it has been commented above, a greater use of solvents will require more energy expenditure to recover the solvents used. It would also be interesting to know how the different extraction equipment and the sample and solvent configurations influence the compounds that make up the rubber and resin, especially those contained in the resin such as guayulins and argentatins.

## 3. Materials and Methods

### 3.1. Plant Materials

The plant material came from an established 16 month guayule field (planted in 2019) in Santa Cruz de la Zarza (Toledo, Spain). This area has dry climatic conditions suitable for guayule cultivation. Average temperature in winter = 6.5 °C, in summer = 22.5 °C; total annual rainfall = 400 mm, with 3 dry months (Pmm/T °C < 2). A total of 150 samples of the following accessions (CAL-1, CAL-2, CAL 7, AZ-2, AZ-3, AZ-5, A 48118, N 565, 11600, 11693, R1040) were generated for the experiments on guayule sample preparation. Each sample was produced by randomly removing four lateral stems from four adjacent plants of the same accession. The samples were stored in kraft paper bags that were opened at the time they were put in the drying oven, where they were dried for 48 h at 60 °C to achieve a moisture content <12%, and then leaves were manually removed from the stem. The stems were cut into pieces of about 1 cm in length using a manual cutter and ground in the following two steps. Firstly, 2 mm size particles were obtained with a 2 mm hammer grinder and secondly 0.5 mm size particles were obtained with a 0.5 mm centrifuge grinder (Retsch centrifugal Mill ZM1, Haan, Germany). Both are continuous flow mills with a sample residence time of fewer than 30 s to avoid heating. Dried ground samples of stems were stored in screw-cap glass vessels at 4 °C until analysis.

For experiments designed to optimize the number of cycles and duration time, three larger samples from two different accessions (AZ-2 and CAL-1) were harvested. Twelve whole plants were harvested from each accession and reduced to 0.5 mm particles following the same protocol as described in the previous paragraph. For the AZ-2 accession, two samples were collected from two different fields to assess whether location may affect their behavior during the extraction process (seeds from the same lot were used, and the plots were planted at the same time).

### 3.2. Optimization of BÜCHI E-914 Accelerated Solvent Extraction for Resins and Natural Rubber Quantification

Extractions were carried out in a BÜCHI E-914 Speed Extractor (Postfach, Switzerland) using a cellulose acetate filter (BÜCHI, Postfach, Switzerland) placed at the top and bottom of each cell to retain the sample and avoid sample cross-contamination. A sequential process was used in which, firstly, the resin was extracted with acetone (Sigma-Aldrich, St. Louis, MO, USA) at 40 °C and 100 bar pressure and, secondly, the natural rubber was extracted with hexane (Honeywell Riedel-de Häen, Charlotte, NC, USA at 120 °C and 100 bar. Other fixed parameters during the extraction procedures were the following: 3 extraction cycles, 1 min heat-up, 3 min discharge, 2 min flush with solvent, and 5 min flush with N_2_. The extraction parameters for optimization were as follows: the amount of guayule (3.0, 1.5, 1.0 g ± 0.005 g), sand as packing material (16.0/16.0, 23.4/23.4, 9.0/12.0, 37.5/37.5 g), sand as a dispersing agent (9.0, 4.5, 32.5, 32.5 g), extraction cell volume (40 and 80 mL), and cycle time (5, 10, 20, 30 min).

Resin and rubber extracts were collected in 250 mL vials and transferred into pre-weighed solvent evaporation flasks that were previously in the desiccator for 30 min. Solvent evaporation was carried out in a Multivapor BÜCHI P-6 parallel evaporator (Postfach, Switzerland) at 50 °C and 150 mbar. The flasks were stored for 30 min in a desiccator before weighing. The resin or natural rubber percentage was then determined gravimetrically. Each sample was extracted twice.

### 3.3. Data Analysis

Two different sets of experiments were performed for adapting the ASE method for resin and rubber determination with the BÜCHI Speed Extractor. The first one, which focused on guayule sample preparation and equipment adjustment, was performed with 150 samples from 11 different accessions. The coefficients of variation (CV), calculated as (standard deviation/mean) × 100, were analyzed to compare the repeatability of the conditions assayed. All of them were represented as boxplots on the basis of the minimum, the 1st and 3rd quartile, the median, and the maximum extraction values for both resins and rubber.

Multivariate analysis was conducted to adjust the cycle times in the second experiment and was used to calculate the variability within each cycle (1st, 2nd, and 3rd) and extraction schemes (5/25/30, 10/20/30, and 20/20/20) for a percentage of extracted resin and rubber in the different accessions (AZ-2a, AZ-2b, and CAL1). Finally, analysis of variance (ANOVA) was conducted to compare the evolution of resins and rubber extraction in time for the three accessions (AZ-2a, AZ-2b, and CAL1). Statistical analysis was performed with IBM SPSS Statistics v25 (IBM Corp., Armonk, NY, USA).

## 4. Conclusions

Accurate and rapid quantification of the resin and rubber content of guayule shrub is needed to accelerate breeding and selection efforts in support of the developing guayule industry. At an industrial level, a method that allows simple and fast quality control tests for quick decision-making will also be needed. The BÜCHI E-914 equipment can quantify resins using only two extractions of five mins each; two additional cycles of 20 min each are sufficient for rubber quantification reducing the overall extraction time compared to published Dionex ASE protocols. The optimal sample preparation method is 1 g guayule (0.5 mm particle size) dispersed in 32.5 g of sand in an 80 mL cell, using a sandwich conformation with sand above and below the guayule/sand sample mixture. An international inter method assay comparison among different laboratories with ASE equipment of different designs and technical configuration would be very useful in setting accurate protocols for guayule resin and rubber quantification.

## Data Availability

The data presented in this study are available on request from the corresponding author.

## Sample Availability

Samples of the compounds are not available from the authors.
